# The Role of Hypoxia in Glioblastoma Invasion

**DOI:** 10.3390/cells6040045

**Published:** 2017-11-22

**Authors:** Ana Rita Monteiro, Richard Hill, Geoffrey J. Pilkington, Patrícia A. Madureira

**Affiliations:** 1 Centre for Biomedical Research (CBMR), University of Algarve, Campus of Gambelas, Building 8, Room 3.4, 8005-139 Faro, Portugal; a50039@ualg.pt; 2 Brain Tumour Research Centre of Excellence, Institute of Biomedical and Biomolecular Sciences, University of Portsmouth, Portsmouth PO1 2DT, UK; richard.hill@port.ac.uk (R.H.); geoff.pilkington@port.ac.uk (G.J.P.)

**Keywords:** GBM, hypoxia, HIF, invasion, chemotherapy

## Abstract

Glioblastoma multiforme (GBM), a grade IV astrocytoma, is the most common and deadly type of primary malignant brain tumor, with a patient’s median survival rate ranging from 15 to 17 months. The current treatment for GBM involves tumor resection surgery based on MRI image analysis, followed by radiotherapy and treatment with temozolomide. However, the gradual development of tumor resistance to temozolomide is frequent in GBM patients leading to subsequent tumor regrowth/relapse. For this reason, the development of more effective therapeutic approaches for GBM is of critical importance. Low tumor oxygenation, also known as hypoxia, constitutes a major concern for GBM patients, since it promotes cancer cell spreading (invasion) into the healthy brain tissue in order to evade this adverse microenvironment. Tumor invasion not only constitutes a major obstacle to surgery, radiotherapy, and chemotherapy, but it is also the main cause of death in GBM patients. Understanding how hypoxia triggers the GBM cells to become invasive is paramount to developing novel and more effective therapies against this devastating disease. In this review, we will present a comprehensive examination of the available literature focused on investigating how GBM hypoxia triggers an invasive cancer cell phenotype and the role of these invasive proteins in GBM progression.

## 1. Introduction

Glioblastoma multiforme (GBM) is the most common type of primary malignant brain tumor, classified by the World Health Organization (WHO) as a grade IV astrocytoma. Patients’ median survival is low, ranging from 15 to 17 months [1,2,3]. Under the Stupp protocol, the current treatment for GBM commences with tumor resection surgery based on MRI image analysis (if possible depending on the location and size of the tumor), followed by radiotherapy and treatment with temozolomide [4]. Radiotherapy alone can significantly increase median survival, although the most common radiological response is to stabilize the disease but ultimately tumor progression follows. Subgroup analysis of GBM patients demonstrated that a discernible clinical response to temozolomide was principally limited to those tumors containing a specific epigenetic alteration, namely promoter methylation of the O6-methylguanine DNA methyltransferase (*MGMT*) gene [5]. Critically, while these patients show an improvement (compared to surgical resection and radiotherapy alone), almost all demonstrate eventual progressive disease in the absence of MGMT promoter methylation [6]. The development of novel and more effective therapeutic approaches for GBM is paramount, taking into account the low median survival rate for GBM patients even when undergoing the current treatment protocols.

Genomic studies have identified four subtypes of GBM based on gene expression patterns, namely the classical, proneural, neural, and mesenchymal subtypes [7,8]. The classical subtype was shown to present amplification or mutation of the epidermal growth factor receptor (EGFR) and high expression of the neural precursor and stem cell marker NES, as well as of the Notch (NOTCH3, JAG1, and LFNG) and Sonic hedgehog (SMO, GAS1, and GLI2) signaling pathway proteins [8]. The proneural subtype shows amplification of platelet-derived growth factor receptor A (PDGFRA) and deletion of the *TP53* tumor suppressor gene [8]. The proneural signature further contains high expression of several proneural developmental genes, such as *SOX*, *DCX*, *DLL3*, *ASCL1*, and *TCF4* [9]. Interestingly, within this subtype isocitrate dehydrogenase (*IDH*) gene mutations associated with improved patient outcome are also observed [8]. The neural subtype is characterized by the expression of neuron markers such as *NEFL*, *GABRA1*, *SYT1*, and *SLC12A5* [8]. Finally, the mesenchymal subtype as the name indicates expresses mesenchymal markers such as CHI3L1 and MET [9] and has been associated with mutations or deletion of the *neurofibromin 1* (*NF1*) gene [8,10,11]. Neurofibromin protein stimulates RAS intrinsic GTPase activity, thereby promoting the conversion of active RAS-GTP into its inactive RAS-GDP state. Hence, the loss of *NF1* gene results in sustained intracellular levels of active RAS-GTP, subsequently promoting oncogenesis [12]. 

More recently, the WHO updated the classification of the tumors of the Central Nervous System (CNS) using molecular parameters in addition to histology [13]. According to the new WHO classification, CNS tumor diagnoses should consist of the histopathological name followed by the genetic features. This new classification subdivides GBM into GBM, IDH-wildtype (approximately 90% of all cases) and GBM, IDH-mutant. The IDH enzymes are responsible for the oxidative carboxylation of isocitrate to α-ketoglutarate producing nicotinamide adenine dinucleotide phosphate (NADPH). Mutations in the *IDH* genes lead to an approximately 50% decrease of the normal IDH activity. Taking into account the impaired function of the mitochondria in GBM cells, the production of bioenergy and intermediates is decreased in IDH mutant GBM, hence the tumor growth is impaired when compared to IDH-wildtype GBM, which translates into a better overall patient prognosis [14].

**Figure 1:**
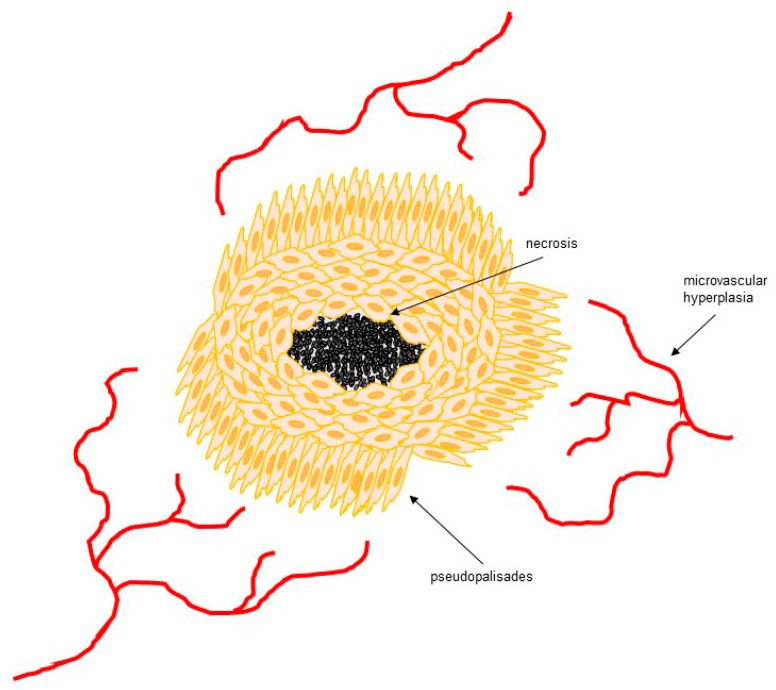
GBM distinctive pathological features. GBM is characterized by necrotic foci with surrounding cellular pseudopalisades and microvascular hyperplasia. Pseudopalisades are created by tumor cells migrating away from a central hypoxic (low oxygenated) region and forming an invasive front. Microvascular hyperplasia is an exacerbated form of angiogenesis that occurs in response to the secretion of proangiogenic factors (e.g., vascular endothelial growth factors (VEGFs), interleukin-8 (IL-8)) by the cells that form the pseudopalisades.

The majority of GBMs develop *de novo* and as such are classified as primary GBMs. These tumors tend to grow rapidly and usually do not have *IDH* mutations. However, a minority of GBMs can slowly develop from low-grade astrocytomas, known as secondary GBMs. Mutations in *IDH* are more frequently observed in this class of GBMs [15]. Histologically, both primary and secondary GBMs are identical. GBM distinctive pathological features include necrotic foci with surrounding cellular pseudopalisades and microvascular hyperplasia (Figure 1), which are believed to play a main role in the accelerated growth and invasion of GBM [16]. Research on the origin of pseudopalisades suggests that this morphological structure is created by tumor cells migrating away from a central hypoxic (poorly oxygenated) region and forming an invasive front [17,18]. Microvascular hyperplasia is an exacerbated form of angiogenesis that occurs in response to the secretion of proangiogenic factors (i.e., vascular endothelial growth factors (VEGFs), interleukin-8 (IL-8)) by the cells that form the pseudopalisades [19]. Microvascular hyperplasia is characterized by a large number of enlarged and rapidly dividing endothelial cells, pericytes, and smooth muscle cells that form tuft micro-aggregates at the leading edge of sprouting blood vessels [20]. It can take the shape of ‘glomeruloid bodies’ that constitute a characteristic feature of GBM. The excessive VEGF production observed in GBM favors the hyper-proliferation and recruitment of endothelial cells in detriment to pericytes that provide coverage and support to the blood vessels [21]. This results in the formation of defective and permeable blood vessels that can easily collapse yielding hypoxic foci within the GBM. Low tumor oxygenation, also known as hypoxia, constitutes a major problem for GBM patients, since it promotes tumor cell spreading (invasion) into the healthy brain tissue in order to evade this adverse environment [22,23]. Tumor invasion not only constitutes a major obstacle to therapy, but it is also the main cause of death in GBM patients. For this reason, an increasing amount of research has been focused on characterizing the molecular and cellular mechanisms that regulate GBM cell invasiveness. Since the hypoxic environment is a major inducer of the GBM cell invasive phenotype, understanding how hypoxia triggers the GBM cells to invade is paramount for the development of novel and more effective therapies against this devastating disease. In this review, we will present a comprehensive examination of the available literature regarding how GBM hypoxia triggers an invasive cancer cell phenotype, the role of these invasive proteins in GBM progression, and whether any of these are therapeutically targetable.

## 2. Hypoxia in GBM

Hypoxia is a condition in which an organism or a cell is deprived of adequate oxygenation (e.g., when oxygen demand exceeds oxygen supply, O_2_ partial pressure (pO_2_) less than 10 mmHg). Hypoxia is frequent in solid tumors, being the natural consequence of the increased oxygen diffusion distance due to tumor expansion. The clinical-pathological effects of hypoxia in GBM can be observed by magnetic resonance imaging (MRI) where significant oxygen diffusion restriction is detected, consistent with absent or defective blood flow [20,24,25]. Molecular markers of hypoxia, such as hypoxia inducible factor 1 (HIF-1) and VEGF staining, as well as tumor vascularity, can be detected on dynamic contrast enhanced MRI and correlate with worse progression-free and overall patient survival [24,26]. Microscopy analyses of GBMs reveal multiple hypoxic regions and a widespread invasion in the growing periphery of these tumors [23]. Hypoxia occurs in GBM due to increased cell proliferation/tumor growth linked to an erratic tumor neovascularization that leads to poor oxygen diffusion [27]. GBM vessels are tortuous, disorganized, highly permeable, and characterized by abnormalities in their endothelial walls due to the lack of pericyte coverage [28,29,30]. These vessels also have significantly larger diameters and thicker basement membranes compared to those of the normal brain [31]. At the tumor tissue level, the occurrence of microvascular thromboses that lead to vessel occlusion is commonly observed, further promoting intratumoral hypoxia [25]. Intratumoral blood flow is thus impeded, creating a heterogeneous tumor environment with respect to oxygenation and interstitial fluid pressure [32]. Inconsistent intratumoral oxygenation leads to hypoxia, acidosis, and necrosis, whereas the increased hydrostatic pressure outside of the GBM vasculature promotes intratumoral edema, a major cause of morbidity for GBM patients [33]. 

## 3. HIF Transcription Factors

HIF transcription factors constitute the master regulators of the hypoxia adaptive response. HIFs are heterodimeric complexes constituted by O_2_-regulated α subunits (HIF-1α, EPAS1/HIF-2α, or HIF-3α) and a constitutively expressed β subunit, HIF-1β, also known as an aryl hydrocarbon receptor nuclear translocator (ARNT) [34,35]. Of the three α subunits, HIF-1α and HIF-2α are the best studied and considered to be the main regulators of the hypoxia response. HIF-1α is ubiquitously expressed, whereas HIF-2α is selectively expressed in distinct cell populations [34]. HIF-1α and HIF-2α have both overlapping and distinct target genes [35] and are differentially regulated under various physiological and pathological conditions [36]. For instance, they play different roles in tumorigenesis depending on specific tumor microenvironments [34,35]. The existence of multiple variants of HIF-3α has made it very challenging to elucidate HIF-3 functions. Distinct HIF-3α variants are expressed in different tissues and are differentially regulated by hypoxia. While the full-length HIF-3α protein has been shown to function as a transcription activator, triggering a unique transcriptional program in response to hypoxia [37], some HIF-3α variants act as dominant-negative regulators of HIF-1/2α functions [38]. The protein structures of HIF-1α, HIF-2α, and HIF-1β subunits contain a basic helix-loop-helix (bHLH), a Per-Arnt-Sim (PAS) domain, and a C-terminal (C-TAD) domain, while HIF-α subunits contain an additional oxygen-dependent degradation domain (ODD) and an N-terminal domain (N-TAD). The bHLH and PAS domains are involved in heterodimer formation and binding to hypoxia responsive elements (HRE) within the promoters of HIF target genes, whereas the N-TAD and C-TAD domains are involved in transactivation through interactions with the transcriptional coactivators p300/CBP [39,40,41]. HIF-3α can exist in many different variants, with distinct deletions of the domains described above (for a detailed description, see [38]). Under normoxic conditions (normal oxygen levels), two prolyl residues within the HIF-α subunits are hydroxylated by prolyl hydroxylases 1-3 (PHD1-3). This allows the binding of the von Hippel-Lindau (VHL) protein to the HIF-α subunit. The VHL protein then recruits E3 ubiquitin ligases, which target HIF-α for proteasomal degradation [42,43,44]. PHDs are 2-oxoglutarate- and iron-dependent dioxygenases, whose activities are positively regulated by O_2_. Hence, hypoxia leads to PHDs inhibition and subsequent stabilization of the HIF-α subunits. HIF-α then translocates to the nucleus and binds to the HIF-1β subunit and cofactors such as p300/CBP; this complex subsequently binds to HRE within promoters of target genes and orchestrates a concerted transcriptional response to hypoxia [45,46,47]. Factor-inhibiting HIF (FIH) is also an O_2_ regulated hydroxylase that blocks the interaction between HIF-α and the transcriptional activators, p300/CBP, through asparagine hydroxylation of HIFα, attenuating HIF transactivation activity [48,49].

**Figure 2:**
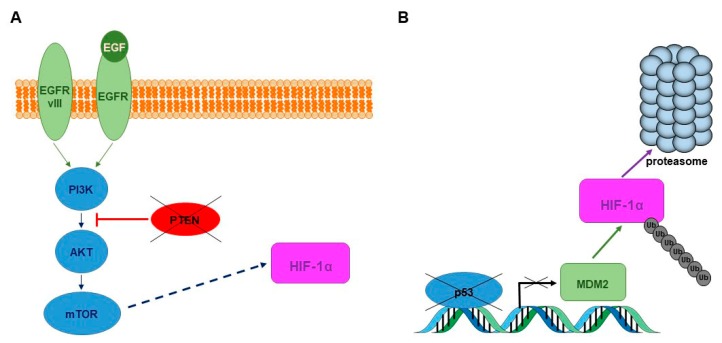
Genetic alterations that lead to HIF activation in GBM. (**A**) *EGFR* gene amplification and/or overexpression is frequent in GBM. The most common *EGFR* gene mutation (EGFRvIII) consists in the deletion of exons 2–7, resulting in a constitutively active and ligand independent receptor. Initiation of EGFR/EGFRvIII signaling by ligand binding, gene amplification, or mutation results in activation of the PI3K/AKT/mTOR pathway with the subsequent up-regulation of HIF-1α. *PTEN* gene deletion is common in GBM. PTEN protein is the main inhibitor of the PI3K/AKT signaling pathway, as such loss of PTEN function leads to increased HIF-1α via the PI3K/AKT/mTOR pathway; (**B**) It has been proposed that p53 may lead to inhibition of HIF activity in hypoxia by promoting MDM2-mediated ubiquitination and degradation of HIF-1α. Therefore, the loss of the *p53* gene, which is common in GBM, will lead to HIF-1α stabilization.

Genetic alterations that lead to HIF activation have been reported in GBM, further exacerbating the hypoxia response in this type of tumor. The activation of the epidermal growth factor receptor (EGFR) and the loss of tumor suppressor function (p53, PTEN) are common in GBM and can affect HIF expression. *EGFR* gene mutation and/or amplification is frequent in GBM [50]. The most common *EGFR* gene mutation (EGFRvIII) consists of the deletion of exons 2–7, resulting in a constitutively active and ligand independent receptor [51]. Initiation of EGFR signaling by ligand binding (which is exacerbated in GBM cells over-expressing EGFR) or gene mutation (i.e., EGFRvIII) results in activation of the PI3K/AKT/mTOR pathway with the subsequent up-regulation of HIF-1α (Figure 2A) [52,53]. GBM has a 20% to 40% incidence of phosphatase and tensin homolog (*PTEN*) deletions [54]. PTEN is the main inhibitor of the PI3K/AKT signaling pathway. Consequently, the loss of PTEN leads to increased HIF-1α via the PI3K/AKT/mTOR pathway (summarized in Figure 2A). It has been proposed that p53 protein may lead to the inhibition of HIF activity in hypoxia by promoting MDM2-mediated ubiquitination and the degradation of HIF-1α [55]. Therefore, the loss of the *p53* gene, which is common in GBM, will lead to HIF-1α stabilization (Figure 2B). Other proteins that have been shown to regulate HIF-1α expression under hypoxic conditions leading to increased GBM cell migration and invasion include FAT atypical cadherin 1, integrins αvβ3 and αvβ5, Hypoxia-inducible protein 2 (HIG2), and geranylgeranyltransferase I (GGTI) [56,57,58,59].

As a result of our understanding of the deregulation of the PI3K/AKT/mTOR signaling pathway in GBM, its targeting has attracted significant attention for the development of anti-cancer therapeutics. Initially, rapamycin analogs (mTOR inhibitors) were tested, although these demonstrated little impact on overall patient survival [60,61]. BKM120 (Novartis) is a pan-class I PI3K inhibitor without mTOR or Vps34 activity that can cross the blood brain barrier (BBB), which is critical for clinical applications against GBM. Preclinical studies demonstrated that the survival of NOD/SCID mice harboring intracerebral U87MG tumor xenografts significantly improved following BKM120 treatment [62]. To date, there is an ongoing phase II clinical trial with this agent in patients with first or second GBM recurrence (clinical trial #NCT01339052) that is being coordinated by the Dana-Farber/Brigham and Women’s Cancer Center, U.S.A. Beyond single agent inhibitors, the dual inhibition of both the PI3K and mTOR pathway is considered a more effective therapeutic strategy, the most extensively studied being BEZ235 (Novartis), a dual PI3K/mTORC1/2 inhibitor [63]. BEZ235 has been used in a wide range of in vitro studies [64] and tested against glioma cells where BEZ235 treatment induced G_1_ cell-cycle arrest and autophagy, and reduced VEGF expression [65]. Furthermore, BEZ235 significantly increased the survival of tumor bearing mice [66]. Recent studies have highlighted conflicting results regarding the effectiveness of this agent in vivo where BEZ235 demonstrated antitumor efficacy with improved survival against U87MG orthotopic gliomas in one study [67]. However, very little to no efficacy was observed in another independent orthotopic xenograft model study [68]. It is important to note that many PI3K/mTOR inhibitors demonstrate weak affinities for ABC transporters, but in spite of this, can still achieve target inhibition in GBM albeit with modest single-agent efficacy. For this reason, it is likely that these drugs will require combination-treatments with other (BBB penetrable) inhibitors and agents.

HIF-induced transcription regulates hundreds of genes that promote angiogenesis, erythropoiesis, migration, cell survival, proliferation, epithelial to mesenchymal transition (EMT), the recruitment of inflammatory cells, invasion, metastasis and metabolic reprogramming, and of particular importance, the shifting of cellular metabolism from oxidative phosphorylation to glycolysis [36,45,69,70,71]. However, the alterations of specific genes and their clinical significance in GBM remain to be better explored. Hypoxia is a main promoter of GBM invasion which is tightly linked with poor patients’ survival and chemoresistance. For this reason, identifying and characterizing hypoxia induced proteins that regulate GBM invasion is key for the development of novel and more effective therapies against this deadly type of tumor.

## 4. Hypoxia Driven Up-Regulation of Invasion Proteins in GBM

In this review, we will perform a thorough examination of the literature focused in identifying hypoxia responsive invasion genes and proteins and characterizing the molecular mechanisms by which these proteins contribute to GBM progression. 

### 4.1. Extracellular Matrix Degradation and Remodeling

Degradation and remodeling of the extracellular matrix (ECM) that surrounds the tumor are essential processes for cancer cell invasion, creating spaces and scaffolding that allow tumor cell proliferation/growth and migration (spreading) away from the initial tumor site [72]. 

Carbonic anhydrase (CA) IX has been shown to be over-expressed in GBM [73,74,75] and *CAIX* gene expression has been shown to be regulated by hypoxia [76]. High expression of CAIX was identified as an independent prognostic factor for poor survival in patients with GBM [77]. An in vitro study using an siRNA approach to knockdown CAIX expression in the GBM cell lines, U251 and Ln 18, showed that CAIX was not only important for GBM cell attachment and invasion, but also for providing radio- and chemo-resistance [77]. CAIX is a membrane bound, zinc dependent enzyme that catalyzes CO_2_ hydration into bicarbonate with a proton release [78]. Since CO_2_ is a main by-product of oxidation, CAs play a main role in maintaining acid/base homeostasis in cells. CAIX has the highest catalytic activity among all membrane bound CAs due to the presence of a unique proteoglycan fragment located close to its active site [79,80]. The CAIX active site is located at the outer side of the plasma membrane, so protons are generated on the extracellular space. The extrusion of hydrogen ions into the extracellular space by CAIX thus contributes to the regulation of the intracellular pH, and at the same time, to the acidification of the extracellular space surrounding the GBM, thus leading to ECM and surrounding tissue degradation and creating an environment conductive to enhanced invasion.

Acetazolamide (a CAIX inhibitor) has been shown to effectively treat 2D or 3D GBM models [81]. These results indicate that CAIX constitutes a promising therapeutic target for GBM. Furthermore, the effectiveness of this agent was significantly enhanced when acetazolamide was incorporated into a polymeric poly(ethylene glycol)-block-poly(lactide-co-glycolide) nano-carrier (that readily crosses the BBB) and used to treat 3D spheroid models [81]. A second CAIX inhibitor, Indisulam, is currently in Phase II of clinical trials for the treatment of a range of cancers, including metastatic melanoma, lung, and metastatic breast cancer where brain metastasis is frequently observed [82].

Epidermal growth factor receptor (EGFR) is amplified in approximately 50% of all GBMs. Expression of the mutant variant of EGFR that lacks exons 2–7 (EGFRvIII), leading to the constitutive activation of this receptor, is particularly common and has been shown to be tightly linked with poor patients’ prognosis [83]. A recent study using U87MG and LN229 GBM cell lines over-expressing EGFRvIII showed that hypoxia stimulates the interaction of EGFRvIII with the integrin β3 in GBM cells, leading to EGFRvIII protein stabilization. Tumor cells adhere to the ECM via integrin receptors which induce the activation of focal adhesion signaling and the subsequent degradation of the ECM, creating a path for cancer cell invasion of adjacent tissues [84]. Interaction of integrin β3 with EGFRvIII stimulated the activation of an integrin β3/SRC/FAK/EGFRvIII signaling axis that led to the activation of ERK1/2 (MAPK), AKT, and STAT3 signaling pathways in the GBM cells and the up-regulation of the cancer cell invasion markers, matrix metalloproteinase-2 (MMP-2) and MMP-9, as well as integrin β3, subsequently promoting GBM cell invasion [85]. 

EGFR is an extremely compelling therapeutic target for GBM treatment. In particular, mutant EGFRvIII [86,87,88], which is reported in approximately 25–33% of all GBM cases [89]. The exclusive expression of EGFRvIII can be targeted by multiple approaches including CAR T-cell therapy, therapeutic vaccines, specific antibodies, or bi-specific T-cell engager approaches, (all extensively reviewed in [90,91]). While there are clinical trials proposed for these agents, there are still significant difficulties waiting to be solved in order to achieve the clinical translation of these agents into GBM patients. These include off-tumor toxicity, potential cross reactivity complications, and a limited tumor penetration capacity. However, the combination of targeting EGFRvIII and other treatment modalities is considered to have significant potential to improve GBM patient prognosis.

Integrins αvβ3 and αvβ5 have also been shown to be recruited to the cell membrane of GBM cells (e.g., U87MG and SF763) in response to hypoxia, leading to the activation of the focal adhesion kinase (FAK) protein to promote tumor invasion [58]. This study also showed that the inhibition of integrins αvβ3 or αvβ5, either by using a specific inhibitor or by siRNA, led to the decrease of HIF-1α intracellular levels. The integrin-dependent HIF-1α regulation was mediated by FAK/RhoB dependent inhibition of GSK3-β which promotes HIF-1α degradation in a VHL-independent manner [92]. 

The proteins of the integrin family constitute promising therapeutic targets. Cilengitide, the most advanced specific integrin inhibitor, has demonstrated anti-tumor activity in both phase I and phase II clinical trials for recurrent GBM [93,94,95]. Treatment of newly diagnosed GBM patients with Cilengitide in combination with conventional therapy (radio-therapy and temozolomide) has been encouraging [96], suggesting synergy of this drug with concomitant chemo- and radiation therapy. A pivotal phase III study (CENTRIC) in newly diagnosed GBM patients was conducted [97]. Within this large phase III study, 3471 GBM patients were screened and 3060 had their *MGMT* status tested. 926 tumours exhibited a methylated *MGMT* promoter, and from this cohort, 545 were randomly assigned to the cilengitide (*N* = 272) or control (*N* = 273) groups. Patient median overall survival was 26.3 months in the cilengitide treatment group and 26.3 months in the control group [97]. Unfortunately, none of the predefined clinical subgroups showed a benefit from cilengitide treatment although the clinical trial did report that there was no overall additional toxic effects with cilengitide treatment [97]. The addition of cilengitide to radiotherapy and temozolomide chemotherapy did not improve patient outcome and the authors state that they will no further develop cilengitide as an anti-GBM drug. Alongside the CENTRIC study, a second independent CORE clinical trial has been conducted (clinical trials identifier NCT00813943). CORE is a Phase II clinical trial for newly diagnosed GBM that recruited 265 GBM patients with the unmethylated *MGMT* gene promoter and incorporated a two-times weekly or an intense, five-times weekly 2000 milligram cilengitide regimen in combination with conventional treatment (temozolomide with concomitant radiation therapy, followed by temozolomide maintenance therapy) for comparison to conventional treatment alone. Temozolomide and radiotherapy treatment alone (*N* = 89) resulted in an overall survival (OS) time of 13.4 months. In comparison, two-time weekly cilengitide treatment (*N* = 88) revealed an OS of 16.3 months. In contrast, intense cilengitide treatment (*N* = 88, at the same 2000 milligram dose) had an overall survival of 14.5 months. When the cilengitide (two-times weekly, in combination with temozolomide and radiotherapy) treatment was compared to temozolomide and radiotherapy alone, and log rank calculated, a *p* value of 0.0328 was reported, indicating that bi-weekly cilengitide alongside conventional treatment significantly improved overall patient survival. It is important to note, that while this CORE study has been completed and data is available (https://clinicaltrials.gov/ct2/show/NCT00813943), this data has not yet been published. Despite the CENTRIC study set back, promising data from the CORE study suggest that the integrins remain potential treatment targets for GBM. 

Procollagenlysine 2-oxoglutarate 5-dioxygenase 2 (PLOD2) is an enzyme that catalyzes collagen cross-linking. A meta-analysis of seven independent GBM datasets, consisting of 861 GBM samples, revealed that *PLOD2* was significantly overexpressed across all GBM datasets analyzed compared to a normal brain [98]. Analysis of *PLOD2* mRNA expression from the Repository of Molecular Brain Neoplasia Data (REMBRANDT) database (*N* = 178), further showed that *PLOD2* mRNA expression was significantly increased in GBM compared to lower grade gliomas and normal brain tissue [98]. Moreover, immunohistochemistry studies demonstrated increased PLOD2 expression associated with increasing tumor grade. Staining for PLOD2 protein was absent or weak in non-neoplastic brain tissue samples and grade II astrocytomas, increased in grade III astrocytomas and enhanced to an even greater extent in GBM [98]. Analysis of The Cancer Research Atlas (TCGA) and the REMBRANDT databases indicated that *PLOD2* can constitute an effective diagnostic marker to distinguish lower grade gliomas from GBM. Moreover, Kaplan-Meier plots based on the TCGA dataset showed significant lower overall survival (OS) and progression free survival (PFS) in GBM patients with high *PLOD2* expression compared to patients with low *PLOD2* [98]. In vitro studies using U87MG and U251 GBM cell lines showed that hypoxia regulates PLOD2 protein expression in a HIF-1α dependent manner and that the knockdown of PLOD2 by shRNA significantly inhibits GBM cell migration and invasion [98]. The molecular mechanism by which PLOD2 contributed to GBM cell migration and invasion involved the phosphorylation of FAK (Tyr 397), leading to increased focal adhesion plaques under hypoxic conditions [98]. Cancer cell invasion is closely related to its ability to stably adhere to the ECM [99] and this process is regulated by a key protein, FAK [100,101]. In vivo studies using subcutaneous and orthotopic xenograft mouse models further showed that PLOD2 promoted cross-linking of collagen fibers around the tumors and enhanced ECM stiffness [98]. The formation of collagen cross-links is initiated by PLOD2, which specifically hydroxylates lysine residues of the collagen telopeptide area [102]. Enhanced ECM stiffness is commonly reported in many types of solid tumors, including gliomas [103,104]. ECM stiffness increases principally as a result of collagen deposition and crosslinking, and this has been shown to induce cancer cell invasion and metastasis [103,104,105]. 

There has been a concerted effort to target PLOD2, particularly in metastatic disease. PLOD2 inhibition not only affects the cancer cells themselves, but it also impacts on the tumor microenvironment, thus impairing metastasis. To date, several pharmacologic inhibitors have been confirmed to have an anti-metastatic effect, while some compounds could be direct PLOD2 inhibitors (reviewed in [106]). Minoxidil (a PLOD2 inhibitor) suppresses the expression of the PLOD2 protein, and significantly reduces cancer cell migration, resulting in an anti-metastatic effect both in vitro and in vivo [107,108]. Berberine can also suppress the expression of PLOD2 and has been shown to inhibit pulmonary metastasis in melanoma [109]. Amentoflavone [110] and beta-carotene [111] also decrease PLOD2 expression, reducing metastasis. Even though there have been substantial advances in targeting PLOD2 in different types of cancer, to date, the effectiveness of these anti-PLOD2 agents in GBM models remains unknown.

### 4.2. Epithelial to Mesenchymal Transition (EMT)

Increasing evidence supports that detachment of cancer cells from the initial tumor site is initiated by the activation of an embryonic development program referred to as epithelial to mesenchymal transition (EMT), whereby epithelial cells lose apicobasal polarity and cell–cell contact and acquire a more motile and invasive mesenchymal phenotype [112].

CBF1 (also known as Recombination signal Binding Protein for immunoglobulin kappa J, RBPJ) constitutes a key transcriptional regulator of the Notch signaling network. The Notch pathway not only regulates stem cell maintenance, but is also a potent inducer of EMT in a variety of tumors [113]. A recent study showed that down regulation of CBF1 expression by shRNA in GBM cells (e.g., GBM1, 407p, JHH) leads to the efficient suppression of EMT activators, including zinc finger E-box-binding protein 1 (ZEB1) protein, *CD44*, and *SNAIL1* genes. CBF1 depletion also correlated with the inhibition of invasion in GBM cells [114], which could be due, at least in part, to the down-regulation of the EMT inducing proteins and genes observed in these cells. To characterize the clinical importance of CBF1, the authors assessed its predictive value by analyzing clinical data sets. This analysis showed that *CBF1* gene expression (assessed by RNA seq) was principally high in pseudopalisades and the peri-necrotic regions of GBM, which are known to be hypoxic areas of the tumor that contain highly invasive cells [114]. Examination of the expression levels of CBF1 in various brain tumor types (as classified by the WHO), showed that GBM has relatively low CBF1 expression [114]. The authors also reported that in two independent data sets (French and TCGA) there was a significant correlation between high CBF1 and patient overall survival; however, this was not observed in another two cohorts (German Glioma Network and Chinese cohort). CBF1 levels were significantly increased in proneural glioblastoma as compared with any other molecular sub-groups as classified by Verhaak et al. [114,115]. Importantly, GBM with mutant IDH1 showed significantly increased *CBF1* expression compared to tumors with wild-type IDH1 [114]. In another study, in silico analysis of the TCGA data set showed that CBF1 was highly expressed in GBM compared to non-neoplastic brain tissue [115]. This study also showed that high expression of CBF1 in *IDH* wild-type GBM correlated with a slightly worse patient’s prognosis [115]. Taking into account these two studies, it is currently still unclear if CBF1 could be a prognostic factor for GBM.

**Figure 3:**
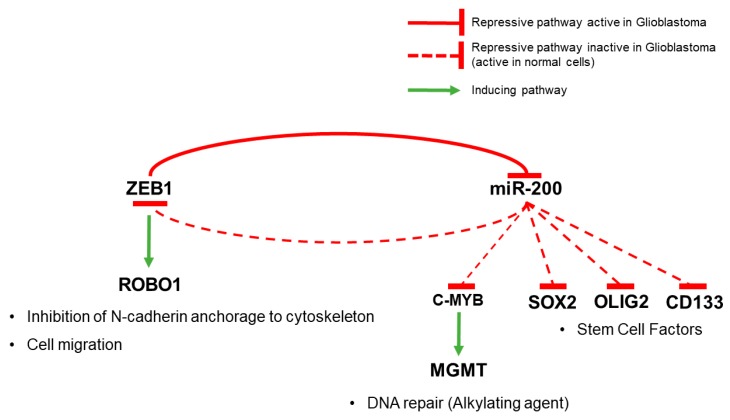
Glioblastoma ZEB1-miRNA-200 feedback loop interactions. In GBM, the ZEB1-miRNA-200 feedback loop targets specific stem cell regulators, namely SOX2, OLIG2, and CD133. ZEB1 up-regulation of c-MYB by the ZEB1-miRNA-200 feedback loop leads to increased expression of the MGMT protein that repairs DNA damage caused by alkylating agents such as temozolomide. ZEB1 positively regulates the ROBO1 protein that has been shown to sever the anchorage of N-cadherin to the cytoskeleton leading to increased GBM cell motility.

Zinc finger E-box binding Homebox 1 (ZEB1) is a transcriptional repressor which has been shown to promote tumor invasion and metastasis [116]. Analysis of the TCGA database showed that ZEB1 is a short survival indicator in GBM patients and a potential therapeutic target [117]. Immunofluorescence and immunohistochemistry studies using human clinical samples showed that ZEB1 is expressed along the tumor invasive front, in pseudopalisades, which are associated with hypoxic areas of the GBM [117,118]. In vitro studies, using a combination of inhibitory drugs and shRNA approaches, showed that hypoxia induces ZEB1 expression and nuclear localization in GBM cells in a HIF-1α dependent manner leading to EMT and increased migration/invasion [118,119]. ZEB1 inhibits the expression of the miRNA-200 family, which suppresses stem cell factors [116]. In GBM, the ZEB1-miRNA-200 feedback loop targets specific stem cell regulators, namely SOX2, OLIG2, and CD133, leading to the activation of the EMT program (Figure 3). Knockdown of ZEB1 in three cell lines generated from primary glioblastoma specimens (hGBM L0, L1, and L2) inhibited invasion [117]. Mechanistically, ZEB1 positively regulated the ROBO1 protein that has been shown to sever the anchorage of N-cadherin to the cytoskeleton leading to increased GBM cell motility and invasion (Figure 3) [117,120]. ZEB1 was also found to play an important role in temozolomide resistance in GBM cells, via up-regulation of c-MYB by the ZEB1-miR-200 feedback loop. c-MYB, in its turn, binds to the *MGMT* promoter in GBM cells, leading to increased expression of the MGMT protein that repairs DNA damage caused by alkylating agents such as temozolomide (Figure 3) [117]. 

TWIST1 transcription factor is also a critical hypoxia induced invasion protein [121]. Physiologically, TWIST1 constitutes a master regulator of embryogenic processes, such as gastrulation and mesoderm differentiation, via the induction of EMT [122,123]. However, TWIST1 has also been involved in pathological processes such as tumor metastasis [121,124,125]. Analysis of *TWIST1* mRNA expression in the publically available databases, REMBRANDT and ATCG, showed that *TWIST1* was more highly expressed in GBM compared to lower grade gliomas [126]. Furthermore, within GBM, the highest *TWIST1* expression was observed in the mesenchymal subtype and lowest expression in the proneural subtype. Elevated *TWIST1* expression was also associated with poor patients’ overall survival [126]. Immunohistochemistry analysis of TWIST1 in 269 infiltrating astrocytoma samples showed a stronger expression of TWIST1 associated with a higher malignancy grade [126]. In addition, positive TWIST1 expression predicted a worse patients’ outcome, which was particularly striking in GBM patients [126]. In vitro studies showed that *TWIST1* mRNA transcription is regulated by tumor hypoxia in a HIF-1α dependent way [127]. The TWIST1 protein was shown to promote an invasive phenotype in GBM cells [128,129], ex vivo using organotypic brain slice cultures and in vivo using a orthotopic xenotransplant mouse model [128]. Nevertheless, TWIST1 did not stimulate the canonical switch between E-cadherin and N-cadherin in GBM cells [128]. Instead, TWIST1 induced changes in cellular interactions (cell-cell and cell-substrate) and cytoskeleton reorganization through alternative pathways in GBM to induce migration and invasion [128], described in detail in Table 1.

### 4.3. Chemokine Receptors

Several studies have shown that C-X-C chemokine receptor type 4 (CXCR4) is highly expressed in GBM, particularly in hypoxic pseudopalisading areas, and plays a critical role in GBM cell invasion [140,141,142,143]. Importantly, no CXCR4 staining was observed in healthy brain tissue [140,141]. These results suggest that CXCR4 expression in GBM might be modulated by hypoxia. In fact, an in vitro study showed that hypoxia leads to the up-regulation of CXCR4 in GBM cell lines (e.g., U87MG and LN308) in a HIF-1α dependent manner [140]. Migration of both U87MG and LN308 cells under hypoxic conditions was significantly increased compared to control normoxic cells and the inhibition of CXCR4 significantly repressed the hypoxia-stimulated migration of GBM cells [140]. CXCR4 is a chemokine receptor for stromal cell-derived factor-1α (SDF-1α), also known as CXCL12. HIF-1 induces SDF-1α expression on hypoxic endothelial cells [144]. Therefore, over-expression of CXCR4 in cancer cells could lead to their migration towards blood vessels.

Inhibiting the CXCR4/SDF-1α axis following local tumor irradiation has shown significant clinical promise and can complement standard GBM treatment. AMD3100 (Plerixafor), an inhibitor of the CXCR4/SDF-1α interaction [145], has been shown to significantly interfere with the vasculogenesis pathway. Treatment of GBM-bearing mice with AMD3100 immediately after irradiation prevented the radiation-induced recruitment of bone marrow–derived cells into the tumor site and critically, AMD3100 exposed tumors could be treated using radio-therapy regimens which were otherwise ineffective [146]. More recently, peptide R, a new specific CXCR4 antagonist, has been developed using a ligand-based screening approach and was shown to impair the metabolic activity and proliferation of U87MG GBM cells. Furthermore, this agent stably reduced CXCR4 expression and cell migration in response to CXCL12 in vitro. Peptide R was shown to reduce tumor cellularity, promote anti-tumor features of glioma-associated microglia/macrophages and astrogliosis, and hinder intra-tumoral vasculature [147]. Overall, CXCR4 targeted therapy has the potential to improve GBM prognosis by supporting conventional therapeutic treatments, as well as potentially treat GBM where standard frontline treatments fail.

C-C chemokine receptor type 5 (*CCR5*) mRNA levels have been shown to be significantly and gradually up-regulated with tumor grade, grade II (*N* = 15), grade III (*N* = 15), and grade IV (*N* = 70) gliomas compared to adjacent non-neoplastic brain tissues [148]. Moreover, immunohistochemistry staining revealed that CCR5 protein expression was gradually stronger with increasing tumor grade [148]. Kaplan–Meier analysis further showed that patients with high levels of *CCR5* expression have significantly shorter disease free survival and overall survival, indicating that *CCR5* may be a potential prognostic marker for GBM [148]. A recent study showed that hypoxia induces CCR5 up-regulation in the GBM cell line, U87MG [149]. Interestingly, hypoxia also enhanced the expression of the CCR5 ligand, CCL4, in THP-1-derived macrophages. Knocking down CCR5 by shRNA in U87MG cells led to a significant decrease in the invasive capability of hypoxic U87MG cells which was concomitant with the decreased transcription of *MMP-9* [149]. Even though these results are interesting and seem to suggest a potentially important role for the CCL4-CCR5 axis in GBM invasion, reproducibility of these experiments using other GBM model cell lines is paramount to strengthening the significance and broadness of these findings.

Interestingly, both CXCR4 and CCR5 chemokine receptors (that have been shown to be up-regulated by hypoxia in GBM), are co-receptors for Human Immunodeficiency Virus 1 (HIV-1). Namely, CXCR4 is the co-receptor for strains that infect T-cells (T-tropic or X4 strains), while CCR5 is the co-receptor for HIV-1 strains that infect macrophages (M-tropic or R5 strains). For this reason, pharmacological drugs targeting these proteins have been developed for the treatment of HIV patients. This is in fact the case for AMD3100 [150] that has been repurposed for cancer therapy as described above.

Studies have also been made to evaluate the use of the FDA approved HIV-1 drug, maraviroc (Pfizer) [151], for the treatment of GBM. Maraviroc is a CCR5 receptor antagonist [152]. Treatment of cancer patients with maraviroc has shown promising results in inhibiting invasion and metastasis in different types of cancer [153,154,155,156]. Unfortunately, to date, the results regarding the use of this drug for GBM treatment are disappointing. A study tested several currently marketed non-chemotherapeutic agents for their ability to enhance/synergize the effect of temozolomide, the current standard chemotherapeutic drug used in the clinic for GBM treatment [157]. This study found that maraviroc (20–180 µM) had no cytotoxic effect against the GBM cell line, GAMG. 

### 4.4. Cytoskeleton Dynamics

Microarray-based gene expression studies using glioma or neuroblastoma cells have shown that knockdown of HIF-1α inhibits the expression of the cyclin G2 encoding gene, *ccng2* [76,158,159]. Furthermore, immunohistochemistry studies showed the expression of cyclin G2 in hypoxic regions of GBM, principally in pseudopalisades [160]. In addition, in vitro studies using U87MG, U251MG, and LNZ308 GBM cell lines showed a significant up-regulation of cyclin G2 expression in response to hypoxia [160]. This study also showed that HIF-1α binds to the *ccng2* promoter and that knockdown of *HIF-1α* by shRNA inhibited the hypoxia-induced expression of cyclin G2. These results indicate that hypoxia is a main regulator of cyclin G2 expression in GBM. Cyclin G2 was first identified as a negative regulator of the cell cycle [161] and later it was shown to induce cell cycle arrest in a p53-dependent manner [162]. Moreover, cyclin G2 was also shown to bind to and stabilize microtubules [162]. A more recent study demonstrated that cyclin G2 plays an important role in the regulation of hypoxia induced migration and invasion in GBM cells (e.g., U87MG and U251MG) [160]. This study revealed that mechanistically cyclin G2 is able to recruit cortactin to the leading edge of migrating GBM cells, promoting the subsequent tyrosine phosphorylation of cortactin, which is essential for ruffle formation and tumor cell invasion [160]. 

### 4.5. Hemostasis

Tissue factor (TF) has been shown to be upregulated in GBM cells in response to hypoxia [25,163,164]. TF is a main regulator of hemostasis, initiating the blood coagulation cascade upon binding to its ligand, factor VII (FVII) [165]. Expression of TF has been shown to positively correlate with the histological grade of gliomas, as well as with the extent of necrosis [166]. A more recent analysis of a large patient cohort of the TCGA data platform (*N* = 424) revealed that *TF* mRNA levels are significantly upregulated in GBM compared to normal brain tissue samples [167]. Kaplan–Meier survival analysis of the REMBRANDT database (*N* = 213) further determined a significant reduction in overall survival in GBM patients with high levels of *TF* expression [167]. Moreover, immunohistochemistry studies revealed that TF is predominantly expressed in perinecrotic and perivascular areas of human GBM [167]. Given the key role of TF in the regulation of blood clotting, increased expression of this protein in tumor cells has been shown to correlate with hyper-coagulation in malignant gliomas, further promoting a hypoxic environment [25,165]. In addition to its pro-thrombotic function, TF has also been shown to constitute a transmembrane receptor that regulates intracellular signaling pathways [165,168,169]. TF/Factor VII (FVII) signaling has been shown to play an important role in glioma cell growth, migration, and invasion [170]. This study suggested that the effects of TF/FVII were mediated through the downstream activation of PAR-2 and the ERK1/2 MAPK signaling pathways. 

There has been some limited research focusing on targeting TF directly (as opposed to ERK 1/2 MAPK inhibitors). A murine xenograft model where human GBM cells of MZ-18 were transplanted into nude mice brains showed that treatment of these mice with a monoclonal antibody against TF (mAb TF9-10H10), using an intracranial osmotic pump system for delivery, significantly inhibited MZ-18 cell invasion compared to mock-treated control animals [167]. The extent of activated blood vessels was also reduced upon anti-TF treatment. These results suggest that targeting TF might be a promising treatment strategy for GBM therapy, by inhibiting both tumor invasion and tumor vasculature. However, clinical trials still need to be performed to test these encouraging results in GBM patients.

### 4.6. Others

Over-expression of the pro-motility receptor protein tyrosine kinase, ephrin type-A receptor 2 (EphA2), has been observed in GBM and associated with poor prognosis/lower overall survival [171,172,173]. In vitro studies have further shown that EphA2 induces GBM cell motility and invasion [174,175,176]. EphA2 activity is antagonized by the EphA2 ligand, ephrin A1 [151,154,155], which is frequently suppressed in GBM, further promoting EphA2 signaling [172,177]. EphA2 coordinates signaling from a variety of receptor tyrosine kinases (RTKs) via growth factor mediated activation of AKT, which initiates AKT-dependent EphA2 phosphorylation at residue S897 [175]. The resulting P-S897-EphA2 is required for lamellipodia formation and subsequent GBM cell motility and invasion [175]. The eHsp90/LRP1 complex was identified as a key regulator of EphA2-dependent GBM cell motility and invasion through its ability to sustain AKT-dependent phosphorylation of EphA2 at residue S897 [178]. Interestingly, hypoxia elicited increased expression and cell surface levels of both eHsp90 and LRP1 in G48a, U87MG, and U251 GBM cells. Moreover, hypoxia-mediated up-regulation of eHsp90 and LRP1 led to amplification of the eHsp90-LRP1 signaling axis, as assessed by robust activation of AKT and EphA2, and concomitantly induced cell motility and invasion [178].

Efforts have been made in the development of HSP90-directed therapies. Several pharmacological inhibitors that target the N-terminal ATP-binding domain of HSP90 have been developed, including ansamycins, purine analogs, and resorcinol derivatives [179,180]. Preclinical and clinical trials have demonstrated the antitumor efficacy of these drugs in different cancer types [180,181]. However, to date, no clinical trials with HSP90 inhibitors have been performed in GBM patients.

Transient receptor potential 6 (TRPC6) mRNA and protein levels have been shown to be up-regulated in GBM samples compared to normal brain tissue [182,183]. TRPC6 is a member of the Transient Receptor Potential (TRP) superfamily of cation channels [184]. In vitro studies using a combination of inhibitory drugs and shRNAs for Notch1 or TRPC6, showed that hypoxia induces the expression of TRPC6 in a Notch1 dependent manner in U373MG GBM cells [182]. Mechanistically, hypoxia induced TRPC6 expression caused a sustained elevation of intracellular calcium levels leading to the activation of the calcineurin-nuclear factor of the activated T-cell (NFAT) signaling pathway and subsequent promotion of GBM cell growth, invasion, and angiogenesis [182]. 

## 5. Conclusions

GBM, the most common and deadly type of brain tumor, is characterized by extensive hypoxic foci that trigger an invasive cancer cell phenotype. Tumor invasion is a major contributor to GBM chemoresistance and patients’ mortality. For this reason, the identification and characterization of proteins involved in hypoxia induced invasion in GBM is critical for the development of novel and more effective therapies against this deadly disease. The treatment protocol for GBM patients has not changed since 2005, and temozolomide treatment in particular has been shown to be primarily limited to tumors with *MGMT* promoter methylation [5]. In this review, we have compiled, to the best of our knowledge, the progress made in identifying hypoxia responsive invasive proteins in GBM, the molecular mechanisms by which these proteins contribute to GBM migration and invasion, and the advancements made regarding the development of pharmacological drugs against these proteins. Proteins involved in ECM degradation and remodeling (e.g., CAIX, integrins β3, αvβ3 and αvβ5, PLOD2, MMP-2, MMP-9); EMT (e.g., CBF1, ZEB1, TWIST1); chemokine receptors (e.g., CXCR4, CCR5); cytoskeleton dynamics (e.g., cyclin G2); hemostasis (e.g., TF, factor VII); the pro-motility receptor, EphA2; and the cation channel protein, TRPC6, have been shown to contribute to GBM migration and invasion in response to hypoxia. Of these, CAIX, integrins, PLOD2, CXCR4, EGFRvIII (that induces up-regulation of MMP-2, MMP-9, and integrin β3 in a HIF-1α/hypoxia dependent manner), HSP90 (which induces hypoxic activation of EphA2) and to a limited extent TF have been shown to be potentially promising targets for GBM treatment. Phase I and II trials are currently being pursued for CAIX, EGFRvIII, PLOD2, and EphA2 targeting chemotherapeutics in different types of cancer. However, clinical trials in GBM patients have not yet been reported for these drugs. Even though a phase III study for the integrins targeting drug, cilengitide (CENTRIC), in newly diagnosed GBM patients showed disappointing results, another independent phase II study with this drug (CORE) showed that bi-weekly treatment with cilengitide alongside conventional therapy significantly improved overall patient survival. While the CENTRIC study was performed in patients with GBM tumors containing a methylated *MGMT* promoter, the CORE study was done in patients with an un-methylated *MGMT* promoter. These results suggest that cilengitide might be beneficial in the treatment of tumors with an un-methylated *MGMT* promoter.

A major obstacle for brain tumor treatment is the BBB. Many of the chemotherapeutics that have shown promising in vitro results against GBM cells do not have the capacity to cross the BBB. For this reason, the development of drug delivery systems that can transport these drugs through the BBB is equally imperative. 

## Figures and Tables

**Table 1:** TWIST1 induced pathways and proteins in GBM.

Protein/Pathway	Role
Periostin	Recruitment of M2 tumor-associated macrophages which are tumor-supportive and immunosuppressive [130].
Fibronectin 1	Promotes cell cohesion and invasion of basement membrane [131].
SPARC	Upholds ECM degradation through the uPA-uPAR pathway and stimulates survival, proliferation and invasion via the PI3K pathway [132].
SNAI2	Inhibits miR-34 activity, a regulatory microRNA for differentiation, promoting stemness [133,134].
ID1	Activates tumor progression pathways, such as Snail, ERK1/2 and AKT, and promotes stem cell self-renewal transcription factors Sox2, Oct3/4 and Nanog [135].
HGF	Promotes survival, proliferation, transformation and invasion through activation of the PI3K/AKT, STAT3/JNK, SOS/RAS/ERK/MAPK pathways [136].
LOX	Activates HIF-1α (via AKT pathway in a positive feed-back loop), *FAK* and *VEGF* gene expression [137].
Cadherin 11	Regulates cell-cell interactions and survival, and promotes cell migration [138].
BMI1/EZH2	Part of the Polycomb repressive complex 1 and 2, in that order, promotes gene silencing of the P16 and P14 tumor suppressors, and inhibits cancer stem cells differentiation [139].
